# Protective Bleaching of Camel Hair in a Neutral Ethanol–Water System

**DOI:** 10.3390/polym10070730

**Published:** 2018-07-03

**Authors:** Liangjun Xia, Chunhua Zhang, Wenfang Xu, Kundi Zhu, Aming Wang, Ye Tian, Yunli Wang, Weilin Xu

**Affiliations:** 1 State Key Laboratory of New Textile Materials and Advanced Processing Technologies, Wuhan Textile University, Wuhan 430200, China; xiali@deakin.edu.au (L.X.); chua_zhang@163.com (C.Z.); wenfang_Xu@163.com (W.X.); zhukundi1@163.com (K.Z.); wangaming0328@163.com (A.W.); 2 Institute for Frontier Materials, Deakin University, Geelong, Victoria 3216, Australia; 3 Guangdong Esquel Textiles Co., Ltd., Esquel Group, Foshan 528500, China; tianye@esquel.com; 4 College of Chemistry and Chemical Engineering, Wuhan Textile University, Wuhan 430200, China; 5 Hubei Biomass Fibers and Eco-Dyeing & Finishing Key Laboratory, Wuhan Textile University, Wuhan 430200, China

**Keywords:** camel hair, natural fiber, bleaching, ethanol–water mixture, whiteness, mechanical properties

## Abstract

As conventional bleaching under alkaline conditions is chemically damaging to protein fibers, a three-stage protective bleaching process in neutral ethanol–water mixtures was proposed for camel hair using mordanting with ferrous salts, oxidative bleaching with hydrogen peroxide, and reductive bleaching with sodium hydrosulfite. The aim of this work was to improve the whiteness degree of camel hair without substantial tenacity loss. In addition, the roles of ethanol during the bleaching treatment were also examined by characterizing the fibers using scanning electron microscopy (SEM), transmission electron microscopy (TEM), Fourier transform infrared (FTIR) spectroscopy, and X-ray diffraction. The whiteness degree and mechanical properties of camel hair bleached in the neutral ethanol–water system were significantly superior to those of fibers bleached by a conventional method. SEM images showed no visible cracks on the scales of fibers bleached in the ethanol–water system, whereas large grooves were observed on fibers bleached in aqueous solution. TEM images confirmed the positive influence of ethanol on the mordanting process, and FTIR spectra suggested that ethanol reduced the breakage of hydrogen bonds in the fibers during the oxidative bleaching process. These findings indicate the potential of this protective bleaching method for application to a broad range of other natural protein fibers.

## 1. Introduction

As an important class of specialty natural fiber, camel hair has distinctive characteristics, such as luster, softness, warmth, and natural colour [1,2,3]. Owing to its exceptional temperature-regulating properties, camel hair is an ideal material for apparel applications. Therefore, the demand for such rare animal fibers may increase as their use in some consumer items, such as high-grade fabrics, makes them more attractive [4]. Despite its small quantitative contribution, the significance of camel hair in the apparel and textile industry should not be underestimated. Camel hair is normally found in various shades of brown or gray; however, high levels of whiteness are essential for apparel fibers [5]. To achieve white or pastel colours, these fibers must be bleached to selectively decolorize the natural pigment before dyeing [6,7]. Hence, investigations of protein fiber bleaching are of practical as well as academic interest.

Several methods are known for protein fiber bleaching, and alkaline hydrogen peroxide has become one of the most widely used bleaching agents for protein fibers, such as wool, cashmere, yak hair, and camel hair. The typical process involves pretreatment with a mordant followed by bleaching with hydrogen peroxide under alkaline conditions [6]. Chen et al. [8] studied the influence of chlorination followed by mordanting with Fe^2+^ and bleaching with hydrogen peroxide on the properties of bleached karakul wool. This modification process was found to improve the felting propensity of karakul wool slightly while remarkably increasing the whiteness. In another study, Mortazavi et al. [9] suggested that the use of Cu^2+^ as a catalyst in the mordanting process before peroxide bleaching enhances the optical properties of karakul wool fibers. This finding was shown more systematically by Yan et al. [10], who explored the relationship between the use of hydrogen peroxide and the properties of protein fibers, showing that the handling and mechanical properties of yak hair improved after bleaching at an optimal concentration of hydrogen peroxide. Interestingly, Arifoglu et al. [11] observed that a high concentration of urea (>100 g/L) plays a significant role in promoting the whiteness of wool and reducing the bleaching time, whereas a lower concentration of urea has no significant effect. In their work on bleaching cashmere, Nakajima et al. [12] suggested that adding small amounts of sodium bisulfite to the rinse bath could effectively promote the whiteness of Mongolian cashmere.

However, as a wet process of the textile industry, bleaching is chemically damaging to protein fibers, especially under alkaline conditions, which can result in considerable loss of breaking tenacity [13,14,15]. The breaking tenacity has an important influence on the finished product rate. ‘Green’ solvents are defined as solvents that have minimal environmental influence arising from their use in chemical production [16]. Hence, to address this issue, alcohols, such as ethanol, have been taken into account. They are nontoxic, environment-friendly, widely available, have low boiling points, and are easily recycled [17,18,19,20]. Additionally, owing to its low surface tension, low viscosity, low molecular weight, and strong permeability, the application of ethanol in textile dyeing has received considerable attention [21,22,23].

Nevertheless, in the past few decades, very little scientific research has been focused on investigating the application of ethanol to bleaching protein fibers, including camel hair. In this work, to obtain maximum whiteness with minimal tenacity damage, we proposed an approach for bleaching camel hair under neutral conditions with the addition of ethanol. Our new method is based on the oxidation-reduction bleaching method and consists of three stages: mordanting with ferrous salts, oxidative bleaching with hydrogen peroxide in mixtures of ethanol–water, and reductive bleaching with sodium hydrosulfite in the aqueous solution. This study was undertaken with the aim of determining a method for improving the whiteness degree of camel hair without substantial tenacity loss.

## 2. Materials and Methods

### 2.1. Materials

Scoured camel hair with a mean diameter of 22.22 μm and an average length of 43.62 mm was kindly supplied by Henan Riyifengda Hair Products Co. Ltd., China. Ferrous sulfate heptahydrate (FeSO_4_·7H_2_O) was used as the mordant. Hydrogen peroxide (H_2_O_2_) was a 30% (*w*/*w*) aqueous solution. Sodium hydrosulfite (Na_2_S_2_O4), absolute ethanol, and all other chemicals were of analytical grade and purchased from Sinopharm Chemical Reagent Co. Ltd., Shanghai, China.

### 2.2. Bleaching

The bleaching process for camel hair mainly consisted of three stages: mordanting with FeSO_4_·7H_2_O, oxidative bleaching with H_2_O_2_, and reductive bleaching with Na_2_S_2_O_4_. All trials were carried out in an XH-KG55B (Foshan Automation Equipment Co. Ltd., Foshan, China) laboratory dyeing apparatus at a fiber-to-liquor ratio of 1:100, and cold deionized water was used for washing fibers throughout the experiments. A more detailed description of each of the bleaching stages follows.

#### 2.2.1. Mordanting

The composition of the ethanol–water mordant bath was 2 g/L of FeSO_4_·7H_2_O in 40% deionized water and 60% absolute ethanol by volume. Camel hair fibers (1.0 g) were introduced into the bath at 55 °C and mordanting was carried out at this temperature for 30 min. After mordanting, the fibers were rinsed at room temperature for at least 10 min and then wrung to 100% water content.

#### 2.2.2. Oxidative Bleaching

Oxidative bleaching was performed in a mixed solution containing 20 g/L of hydrogen peroxide in 100% absolute ethanol. The camel hair fibers were immersed into the bath at 65 °C and treated for 1 h followed by rinsing for at least 10 min. Subsequently, the rinsed fibers were squeezed to remove excess water before being placed into the reductive bleaching bath.

#### 2.2.3. Reductive Bleaching

Reductive bleaching was carried out at 60 °C for 20 min in a bath containing 15 g/L of sodium hydrosulfite in 100% water. The fibers were washed and then dried in air.

**Figure 1:**
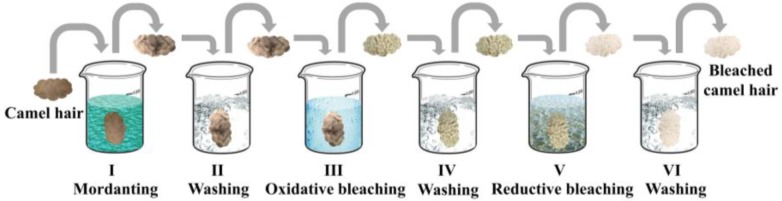
Simulation of experimental procedures.

**Figure 2:**
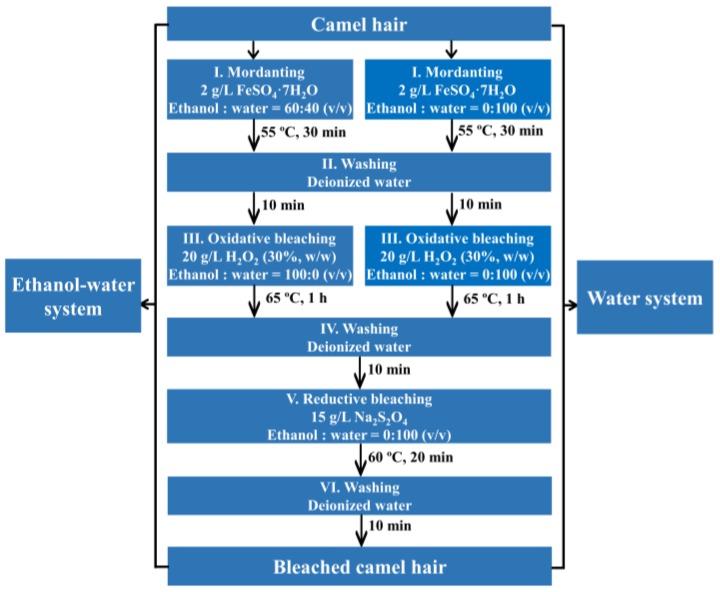
Flow chart for bleaching of camel hair in the ethanol–water and water systems.

Bleaching of camel hair in the water system was carried out under the same conditions, except that only aqueous solutions were used. The experimental procedures and flow chart for bleaching of camel hair in ethanol–water and aqueous systems are shown in Figure 1 and Figure 2, respectively. The optimal conventional bleaching was performed in the aqueous solutions with the addition of desired alkali and chemical auxiliaries.

### 2.3. Characterization

#### 2.3.1. Weight Loss

To evaluate the weight loss after bleaching, the treated sample was weighed and the weight loss percentage of the bleached fibers was evaluated using following equation [24]:

(1) $$Weight\;loss\;\left(\%\right)=\left(m_{1}-m_{2}\right)/m_{1}\times 100\%$$

where *m*_1_ and *m*_2_ are the weight of fibers before and after bleaching treatment, respectively. Before weighing, all samples were dried in an oven at 60 °C until reaching a constant weight.

#### 2.3.2. Degree of Whiteness

The whiteness of all the samples was measured using a whiteness meter (WSB-II, Wenzhou Instruments and Apparatus Co. Ltd., Wenzhou, China). To compare the bleaching treatment in the ethanol–water and water systems, the percentage increase in whiteness was calculated using the following equation [25]:

(2) $$Whiteness\;increase\;\left(\%\right)=\left(w_{1}-w_{2}\right)/w_{2}\times 100\%$$

where *w*_1_ and *w*_2_ are the whiteness of the samples after and before bleaching, respectively.

#### 2.3.3. Mechanical Properties

The mechanical properties of the bleached samples were determined using a Favimat-Airobot 2 system (Textechno H. Stein, Monchengladbach, Germany) at a crosshead speed of 10 mm/min and a gauge length of 20 mm according to ASTM 76. All samples were equilibrated under standard conditions (20 °C, 65% relative humidity (RH)) for at least 48 h before testing. Each sample was tested 100 times and the average values are presented in this paper.

#### 2.3.4. Swelling Ratio

To examine the effect of ethanol on the swelling properties of camel hair, a series of trials were performed. A single fiber was used in each swelling experiment, and its diameter was measured before and after swelling. Each swelling bath was composed of 2 g/L FeSO_4_·7H_2_O in different mixtures of ethanol and water (ethanol:water = 0:100, 60:40, and 80:20 (*v*/*v*)). Then, the single fiber was immersed into the bath at 55 °C and allowed to swell for 30 min. Each single fiber was positioned between two thin glass plates. A standard microscope combined with image analysis software was employed to measure the change in fiber diameter during swelling, and the swelling ratio (*R_s_*) was calculated by the following equation [26]:

(3) $$\;R_{s}\left(\%\right)=D_{a}/D_{b}\times 100\%$$

where *D_a_* and *D_b_* are the average diameter of a fiber after and before swelling, respectively.

#### 2.3.5. Contact Angles

A drop shape analyzer (DSA25, Krüss, Hamburg, Germany) equipped with a video measuring system with a fast and high-resolution USB3.0 camera was used to measure contact angles. To alleviate the effect of droplet deformation on the contact angle resulting from the force of gravity, we chose a drop volume of 2 µL, as this volume maintained its spherical form on the glass slide. The contact angle was measured 1 s after release of the droplet [27], and the contact angles were calculated using the supplied software (Drop shape Analysis, DSA Version 1.92.1.1, Hamburg, Germany).

#### 2.3.6. Scanning Electron Microscopy

The surface morphology of the samples was inspected using scanning electron microscopy (SEM, JSM-IT300, JEOL, Tokyo, Japan) after gold–palladium coating at a voltage of 10 kV with a magnification of 100–3000×.

#### 2.3.7. Transmission Electron Microscopy

The cross-sectional morphologies of the camel hair sample were examined by transmission electron microscopy (TEM, JEOL-2100F, JEOL, Tokyo, Japan) fitted with an energy dispersive X-ray spectroscopy (EDS) detector.

#### 2.3.8. Fourier Transform Infrared Spectroscopy

Fourier transform infrared (FTIR) spectra were recorded using a Vertex 70 spectrometer (Bruker, Karlsruhe, Germany) via reflection-absorption spectroscopy. The data were collected over 128 scans in the range of 500–4000 cm^−1^ with a resolution of 4 cm^−1^. Samples were dried in a vacuum before testing.

#### 2.3.9. X-ray Diffraction

X-ray diffraction (XRD) data was obtained using wide-angle XRD analysis (X’Pert PRO, PANalytical B.V., Almelo, The Netherlands) using Cu Kα radiation at 40 kV and 40 mA. Samples were scanned from 5 to 70° (2θ) with a step size of 0.02°.

## 3. Results and Discussion

### 3.1. Degree of Whiteness and Weight Loss

**Figure 3:**
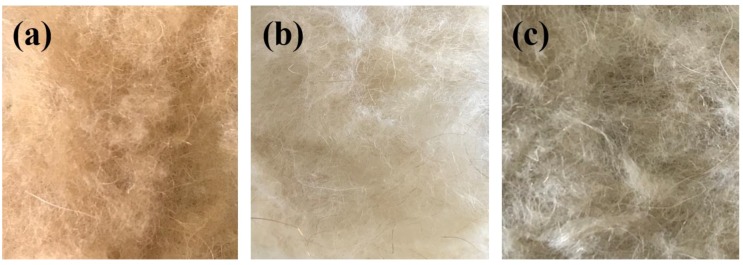
Photographs of control sample (**a**) and samples bleached in the ethanol–water (**b**) and water systems (**c**).

During the bleaching process, the colour of camel hair changes and the underlying whiteness is exposed. To examine the effect of ethanol on the bleaching treatment, photographs were taken and the whiteness and weight loss values of the bleached camel hair were determined as summarized in Figure 3 and Table 1.

As can be seen from Table 1, the degree of whiteness of bleached camel hair was greater than that of the control fibers in all cases. These results indicate that the bleaching treatment in the ethanol–water system was more effective than that in the water system in terms of whiteness and weight loss values. The whiteness of camel hair bleached in aqueous solutions increased from 12.3 to 19.0, whereas that bleached in the ethanol–water system showed a dramatic improvement in whiteness, increasing from 12.3 to 32.4. This difference in whiteness could be ascribed to the deposition of excessive amounts of ferrous ions on the fibers during mordanting in aqueous solution [6]. Additionally, compared with the slight decrease in weight observed for the fibers bleached in the water system, more evident decreases of weight were observed when ethanol was present during the mordanting and oxidative bleaching processes. This increased weight loss may be due to the removal of more pigments and impurities from camel hair during the ethanol-assisted bleaching treatment [28]. Furthermore, the effect of ethanol was evaluated according to the whiteness increase calculated using Equation (2). The obtained whiteness increase values indicate that a specific concentration of ethanol may be important for promoting an effective bleaching process. After bleaching, a nearly 70% higher degree of whiteness was achieved in the presence of ethanol than without the addition of ethanol, indicating that more melanin is removed from the fibers when bleaching occurs in the ethanol–water mixture. In view of the findings of Laxer and Whewell [29], we consider that under the mordanting conditions in the ethanol–water mixture, it may be that only melanin reacts with ferrous ions, whereas keratin does not absorb ferrous ions from the solution.

### 3.2. Mechanical Properties

In order to study the impact of bleaching treatment on the mechanical properties of camel hair, the breaking tenacity, minimum and maximum breaking tenacity, and breaking elongation of different samples were determined as presented in Table 2.

It is apparent that camel hair can be chemically attacked by the mordanting and bleaching agents in both the water and ethanol–water systems. The overall results clearly confirm that the bleaching in the water system caused excessive damage to the bleached camel hair, while the damage incurred by the fibers during the bleaching treatment was significantly reduced by adding ethanol to the baths (Table 2). In particular, the breaking tenacity and breaking elongation values resulting from bleaching in aqueous solution were dramatically lower than those resulting from bleaching in the ethanol–water mixture. The breaking tenacity and elongation at break of the camel hair bleached in the water system decreased from 1.65 to 0.84 cN/dtex and from 36.18% to 16.74%, respectively, indicating that severe damage occurs under these conditions. By contrast, the breaking tenacity and elongation at break of the fibers bleached in the presence of ethanol only exhibited a slight decrease, from 1.65 to 1.24 cN/dtex and from 36.18% to 30.53%, respectively. Additionally, the camel hair bleached in the ethanol–water mixture had a much higher breaking energy and modulus than the fibers bleached in aqueous solution.

**Figure 4:**
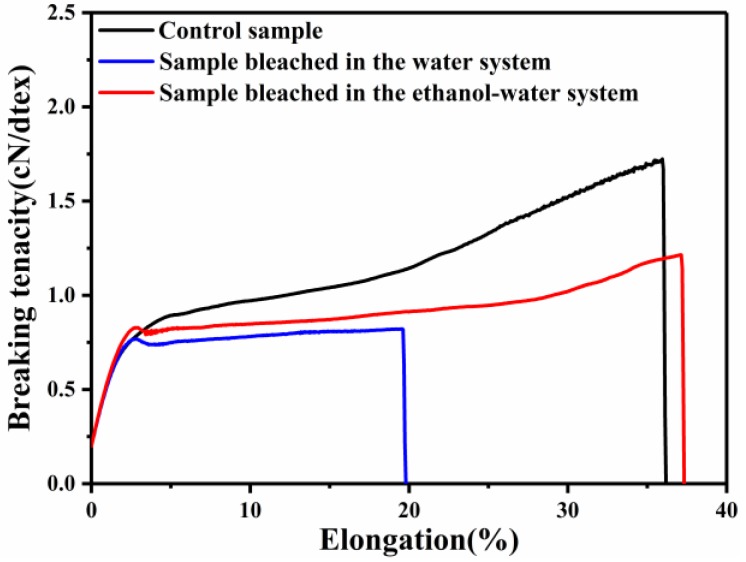
Tenacity-elongation curves of camel hair before and after bleaching treatment.

The tenacity-elongation curves of camel hair before and after bleaching treatment are shown in Figure 4. Compared with the fibers bleached in the water system, the camel hair bleached in the ethanol–water system exhibits a higher tenacity and breaking elongation. According to the investigation of Xiao et al. [30], tensile curves exhibit three distinct phases: a linear Hookean region at strains less than 3%, a yield region at strains from 3% to 25%–30%, and a post-yield region at strains beyond 30%. However, for the camel hair bleached in aqueous solution, the increased stiffness in the post-yield tensile region disappeared.

**Figure 5:**
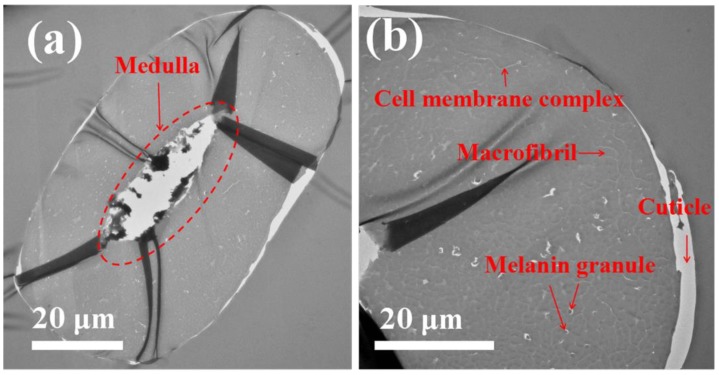
Transmission electron micrographs (TEM) of camel hair. (**a**) A cross section showing the centrally located medulla. (**b**) A highly magnified image showing its arrangement in an irregular form with the cuticle, matrix, macrofibril, and melanin granule.

TEM images of transverse fiber sections show the morphology of camel hair under different magnifications (Figure 5). The centrally located medulla can be clearly observed, which is considered to provide thermoregulatory properties to camel hair. At high magnification, the cuticle, cell membrane complex, and macrofibril structures are well-preserved. Moreover, melanin granules are also observed to be spread irregularly within the fiber.

It is well-known that the tensile properties of camel hair are determined by the fibrils and matrix [30]. Therefore, we consider that the disappearance of the post-yield tensile region in the camel hair bleached in the water system may be related to serious damage of the fibrils and matrix [31]. This would lead to the reduction of disulfide bonds and interfaces between the fibrils and matrix, as the fibrils are assumed to be connected mechanically to the matrix via a number of covalent linkages at various intervals along the axis of the fibrils [32,33].

Furthermore, water is thought to have a considerable effect on the matrix proteins in the fibers, which may contribute to reducing the interactions between protein chains [34,35,36]. Further, in protein fibers, both the –NH and –C=O groups of amides are able to form hydrogen bond interactions in polypeptide chains, both as inter- or intra- chain bonds [37]. As the intrinsic stability of the α-helix, and even the fiber, results from intramolecular hydrogen bonds [38], the attack of hydrogen bonds by hydrogen peroxide may reduce the mechanical properties of camel hair.

This suggestion was confirmed by FTIR analysis, which showed that considerably more hydrogen bonds were broken in the fibers bleached in the water system than in the ethanol-assisted bleached camel hair. Finally, a study by Feughelman [37] suggested that the presence of cysteine in the protein fibers is mainly responsible for the high stability of the fibers during degradation.

### 3.3. SEM

**Figure 6:**
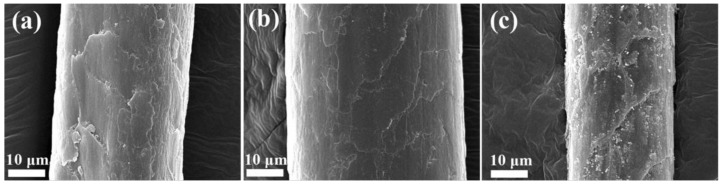
SEM images of camel hair: (**a**) control; (**b**) bleached in the ethanol–water system; (**c**) bleached in the water system.

SEM was employed to observe the surface morphology of camel hair. Figure 6 shows SEM images of the control sample as well as camel hair bleached in the ethanol–water and water systems. In the SEM image of the fibers bleached in the ethanol–water system (Figure 6b), no cracks are visible on the scales of the fibers, meaning that the impact of degradation could be controlled with the addition of ethanol. In contrast, the fibers bleached in aqueous solution (Figure 6c) obviously suffered considerable damage. This serious damage may result from the fact that hydrogen peroxide has an oxidative effect on the surface scales of camel hair. During oxidation, disulfide linkages of cystine, peptide linkages, and hydrogen bonds may be attacked by hydrogen peroxide, resulting in breakage of these interactions [39]. These results confirm that bleaching in the presence of ethanol under neutral conditions makes camel hair less accessible to the proteolytic attack, which is in accord with the results for the mechanical properties of camel hair.

### 3.4. FTIR

**Figure 7:**
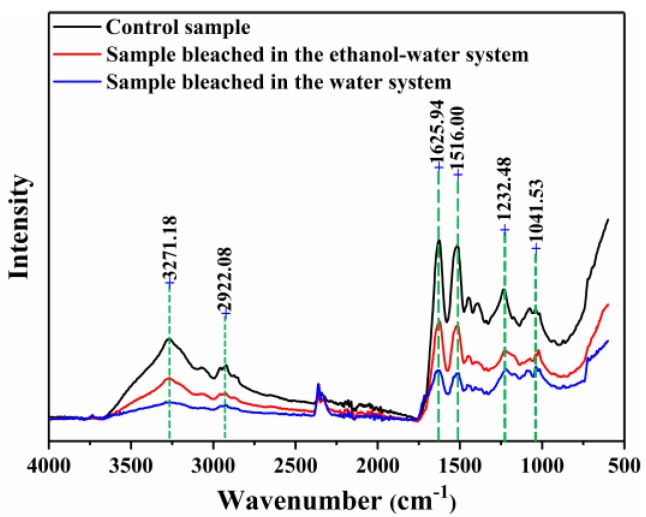
FTIR spectra of camel hair before and after bleaching treatment.

FTIR spectra of camel hair before and after bleaching treatment are shown in Figure 7. Similar absorption bands are observed in the spectra of the three samples at around 3271 cm^−1^ (O-H and N-H), 2922 cm^−1^ (-CH_2_), 1626 cm^−1^ (Amide I), 1516 cm^−1^ (Amide II), and 1232 cm^−1^ (Amide III). These are the typical absorption peaks of protein fibers according to previous investigations [39,40]. No new chemical groups or free residues were formed by the bleaching treatment. However, the peak around 3270 cm^−1^ for the unbleached fibers is sharper than that of the fibers bleached in both the water and ethanol–water systems.

The main structural units in camel hair are successive α-helix turns, which are the largest class of protein secondary structures [41]. According to the work of Hameed and Guo [42], in the secondary structure of proteins, N-H groups in the α-helix are generally hydrogen bonded with C=O groups of the amino acids. Hence, the peak at 3270 cm^−1^ can be attributed to this hydrogen bonding interaction. A greater reduction in the intensity of this peak was observed for the fibers bleached in the water system than those bleached in the ethanol–water system, indicating that the breakage of these intramolecular hydrogen bonds during the bleaching treatment in aqueous solution was comparatively severe.

Similarly, the peaks around 1626, 1516, and 1232 cm^−1^ are sharper for the fibers bleached in the ethanol–water system than for the fibers bleached in the water system, which could be attributed to less damage to some amide groups during the bleaching treatment [39]. The SEM images show that the cuticle of camel hair bleached in the aqueous solution was seriously damaged. These findings are consistent with the changes observed in the mechanical properties of camel hair.

Typically, in the range from 1300 to 1000 cm^−1^, spectra of such fibers are characterized by the presence of medium-to-high intensity bands attributed to various sulfur-containing chemical groups [40]. Cross-linkages in α-keratin fibers are formed by –S-S– groups that contribute to the physical and mechanical properties, as well as the structural stability, of camel hair [43]. Accordingly, the dramatic decrease in the mechanical properties and the destruction of the fibers bleached in the conventional water system could also be ascribed to breakage of these disulfide bonds [44,45].

### 3.5. XRD

**Figure 8:**
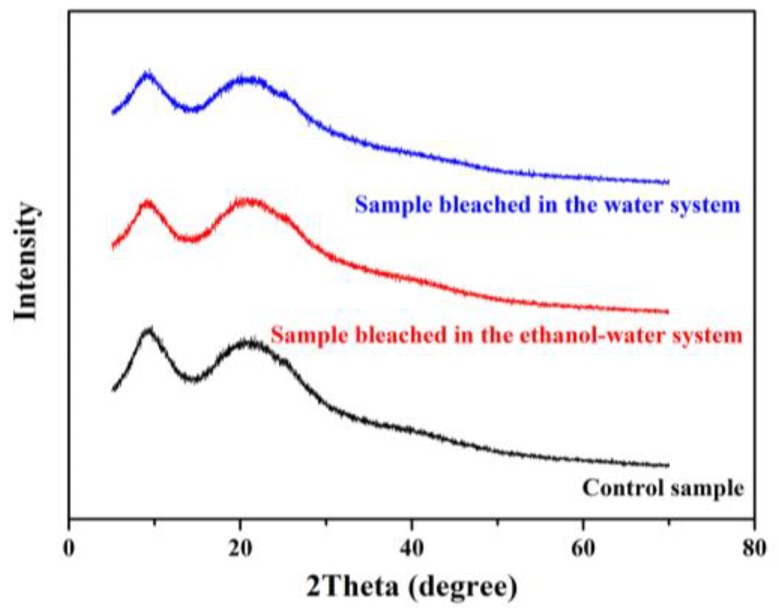
X-ray diffraction intensity curves of camel hair samples.

Figure 8 displays the XRD patterns of the camel hair samples. All the samples show the typical diffraction pattern of α-keratin with a prominent 2θ peak around 10° and a broad peak around 22°, corresponding to crystalline spacings of 9.82 and 4.39 Å, respectively [46]. Compared with the control sample, the intensity of these peaks was decreased after the bleaching treatment. This change may be attributed to the breakage of hydrogen bonds and covalent interactions during the bleaching process, leading to the destruction of some crystals and amorphous regions [40,47]. As the peak around 10° is characteristic of the hydrated crystalline structure of camel hair, the decreased intensity suggested that the α-helix crystal structure was weakened by the bleaching treatment. Moreover, it is possible that the margins of the α-helical crystalline phase were disordered by hydrogen peroxide, resulting in reduced peak intensities.

### 3.6. Effects of Ethanol on Mordanting

To examine the effect of ethanol on the mordanting process, a comparison was made between the camel hair samples mordanted in the water and ethanol–water systems. Camel hair was mordanted in a FeSO_4_ solution at a concentration of 2 g/L in water (100%) or ethanol–water (60%/40%, *v*/*v*) for 30 min at 55 °C for 30 min. Subsequently, the rinsed and dried samples were examined by TEM.

**Figure 9:**
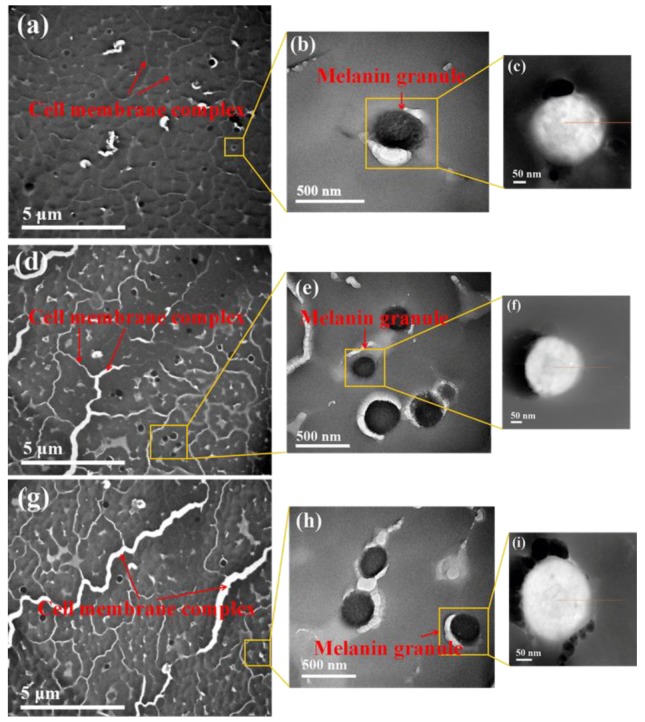
TEM images of camel hair under different magnifications. (**a**–**c**): control sample; (**d**–**f**): sample mordanted in the ethanol–water system; (**g**–**i**): sample mordanted in the water system.

Figure 9 presents TEM images of the cross sections of unmordanted (a–c) and camel hair mordanted in the ethanol–water (d–f) and water (g–i) systems under different magnifications. A comparison of Figure 9a,d,g reveals that the cell membrane complex of the control sample undergoes some kind of modification in the samples mordanted in both the water and ethanol–water systems, probably owing to the swelling of camel hair during the mordanting process with ferrous ions [48,49]. At a higher magnification (Figure 9b,e,h), spherical melanin granules are apparent in the cortical cells.

**Figure 10:**
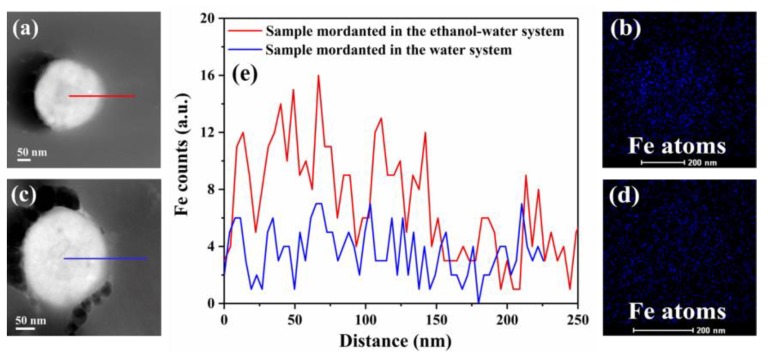
TEM images and EDS elemental mappings. (**a**) TEM images of a melanin granule mordanted in the ethanol–water system; (**b**) EDS elemental mapping of Fe in a melanin granule (**a**); (**c**) TEM images of a melanin granule mordanted in the water system; (**d**) EDS elemental mapping of Fe in a melanin granule (**c**); (**e**) EDS line scan profiles of Fe recorded along the line shown in (**a**,**c**).

It is common knowledge that the chelating activity of melanin for ferrous ions is relatively strong [50]. Hence, to further investigate the influence of ethanol on the adsorption of ferrous ions on melanin granules, elemental mappings of the melanin granules mordanted in the ethanol–water and water systems were obtained using the TEM-EDS technique as shown in Figure 10.

The density of an element is indicated by the relative brightness, and the intensity of colour indicates its distribution in the melanin granules [51,52]. It is clearly observed that the distribution of Fe in the melanin granule mordanted in the ethanol–water system (Figure 10b) is more uniform than that mordanted in the water system (Figure 10d). This was further confirmed by the EDS line scan profiles (Figure 10e), which manifested the existence and relatively uniform distribution of Fe in the melanin granule mordanted in the ethanol–water system. This is mainly due to the strong permeability of ethanol into camel hair. This even distribution may lead to greater catalytic decomposition of hydrogen peroxide in the subsequent bleaching process, resulting in the higher degree of whiteness for bleached camel hair.

**Figure 11:**
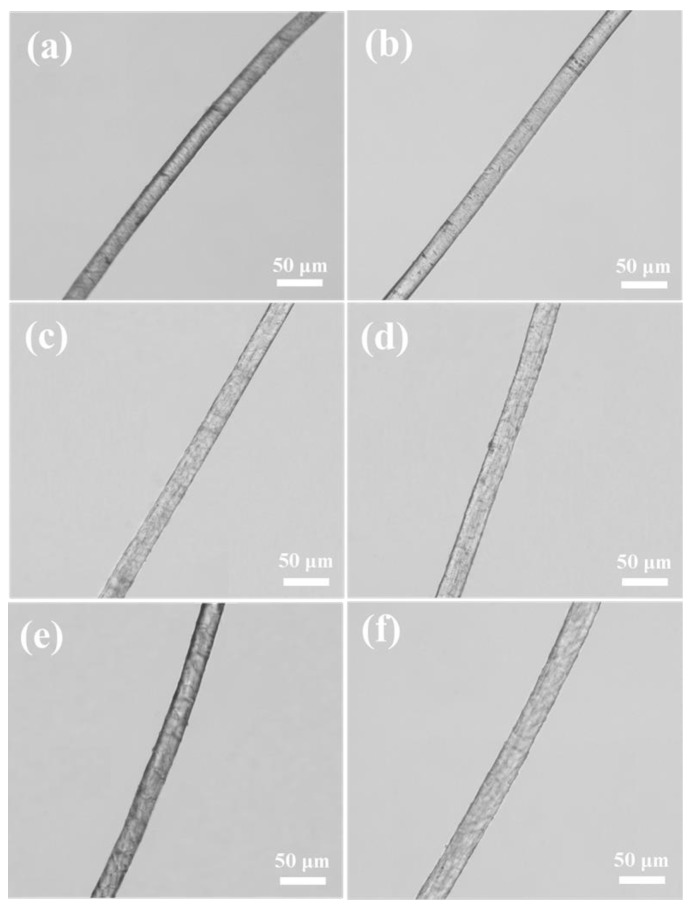
Optical measurements of camel hair diameter before and after swelling in the water and ethanol–water systems: (**a**) Ethanol:water = 0:100 (*v*/*v*), before swelling; (**b**) Ethanol:water = 0:100 (*v*/*v*), after swelling; (**c**) Ethanol:water = 60:40 (*v*/*v*), before swelling; (**d**) Ethanol:water = 60:40 (*v*/*v*), after swelling; (**e**) Ethanol:water = 80:20 (*v*/*v*), before swelling; (**f**) Ethanol:water = 80:20 (*v*/*v*), after swelling.

Then, a series of trials were carried out to examine the effect of ethanol on the swelling properties of camel hair. Wetted length measurements were performed before and after swelling in the water and ethanol–water mixtures using the optical method described in Section 2.3.4. Three swelling tests were conducted on single fibers in the water and ethanol–water mixtures and 10 measurements were taken to obtain the diameters before and after the swelling process as shown in Figure 11.

Table 3 exhibits the average diameters of camel hair before (*D_b_*) and after (*D_a_*) swelling as well as the average swelling ratio (*R_s_*). The presence of ethanol does not dramatically modify the morphology of camel hair, as the surface morphologies of the fibers are similar, but does affect the swelling properties. The swelling of camel hair in the water system and its dispersion were lower than those in different ethanol–water systems, meaning that camel hair was more susceptible to the sorption of ethanol than water, and therefore absorbed more ethanol than water.

The enhanced swelling of fibers in the presence of ethanol may be due to the following reasons. Firstly, as the ratio of ethanol in the mordanting bath increases, the sites in the fiber form more bonds with ethanol; thus, the volume of ethanol absorbed is greater than that of water. Secondly, the fiber, especially α-keratin, forms stronger hydrogen bonds with ethanol than with water; hence, increasing the impetus for swelling of the fiber with ethanol. Thirdly, the nonpolar groups in α-keratin are more likely to form hydrophobic bonds in aqueous solution, leading to decreased absorption of water [37,53].

Furthermore, when examining swelling, especially the effective wetting properties of a fiber when dipped into a solution, it is essential to determine contact angle values, as the wetting performance of the fibers plays an important role in wet processing. Wettability investigations normally involve the measurement of contact angles as the initial data, indicating the wetting performance when a solid and liquid interact. The contact angle is defined as the angle formed by the intersection of the liquid–solid interface [54,55]. In this experiment, the contact angle between a glass slide and a liquid was used to simulate the contact angle between a camel hair fiber and a liquid.

**Figure 12:**
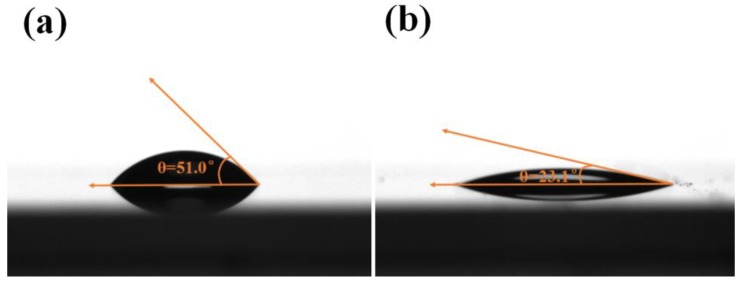
Illustration of contact angles formed by liquid drops on smooth glass slides: (**a**) Ethanol: water = 0:100 (*v*/*v*), 2 g/LFeSO_4_·7H_2_O; (**b**) Ethanol: water = 60:40 (*v*/*v*), 2 g/L FeSO_4_·7H_2_O.

Figure 12 presents the contact angles formed by different liquid drops on smooth glass slides. The liquid drop composed of water and mordant had a contact angle of 51.0°, whereas the contact angle of the liquid drop composed of mordant in 90% ethanol and 10% water by volume was only 23.1°, indicating that the surface tension of the droplet was reduced with the addition of ethanol. It is important to note that the decrease in surface tension may lead to an increase in the affinity between the solution and the fibers [56]. Thus, during the mordanting process, the wetting properties of camel hair in ethanol–water mixtures is greater than that in aqueous solution owing to the reduction in angle contact [54]. As a result, ferrous ions are more likely to be absorbed in the ethanol–water system than in the water system. It is known that ferrous ions have a positive effect on the catalytic decomposition of hydrogen peroxide [57,58,59]. In the classical Fenton-type system, the reaction of ferrous ions with hydrogen peroxide is used to generate hydroxyl radicals, which then carry out chemical oxidation of melanin in camel hair.

Moreover, the mordant used in this study is soluble in water but insoluble in ethanol; hence, the large amount of ethanol acts as a filling agent in the mordanting bath. Consequently, the concentration of Fe(II) ions in the ethanol–water mixture was higher than that in the aqueous solution, which may have had a positive influence on ferrous ion uptake by the camel hair.

On the other hand, the absence of water has been shown to prevent oxidation of Fe(II) to Fe(III) [60]. Accordingly, the concentration of Fe(III) in aqueous solution may be somewhat higher than that in the ethanol–water mixture. Although absorption of Fe(III) may result in a similar mordanting effect as Fe(II), the use of Fe(III) is not as selective, leading to heavy damage of the fiber proteins during the bleaching procedure [61]. Therefore, in the water system, camel hair is more likely to be attacked, which agrees with the observed changes in the mechanical properties of bleached camel hair.

**Figure 13:**
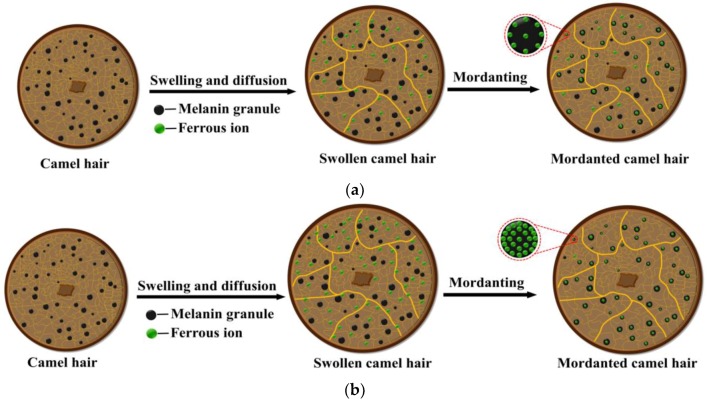
Principle of mordanting in (**a**) the water system and (**b**) the ethanol–water system.

Consequently, based on these findings, the basic principles of the mordanting process in the water and ethanol–water systems are outlined in Figure 13a,b, respectively. In these two systems, camel hair can be swollen in the mordanting process. During the process of swelling and diffusion, ferrous ions are permeated into the swollen camel hair. Then, ferrous ions can be combined with melanin granules in camel hair. However, the camel hair mordanted in the ethanol–water system has a higher swelling ratio compared with that in the water system. Additionally, due to the addition of ethanol, more ferrous ions are permeated into camel hair and then combined with melanin granules. Therefore, the presence of ethanol can benefit the mordanting process greatly, resulting in the absorption of Fe(II) being more selective by the melanin in camel hair.

### 3.7. Effects of Ethanol on Oxidative Bleaching

To investigate the impact of ethanol on oxidative bleaching of camel hair, a series of trials were carried out. Initially, all the samples were immersed in a bath of 2 g/L FeSO_4_·7H_2_O in 60% ethanol and 40% water by volume for 30 min at 55 °C. After this mordanting process, the fibers were rinsed at room temperature for at least 10 min and then wrung to 100% water content. Oxidative bleaching was carried out in a mixed solution of 20 g/L of hydrogen peroxide in different ratios of ethanol and water for 1 h at 65 °C. Subsequently, the samples were rinsed for at least 10 min, dried, and then examined using SEM.

**Figure 14:**
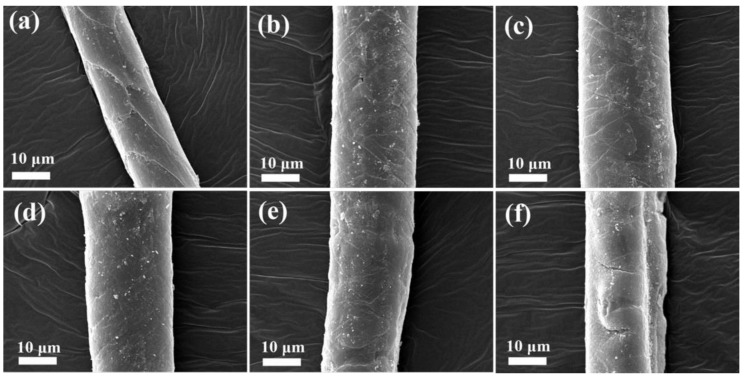
SEM images of bleached camel hair with different contents of ethanol during oxidative bleaching: (**a**) Ethanol:water = 100:0 (*v*/*v*); (**b**) Ethanol:water = 80:20 (*v*/*v*); (**c**) Ethanol:water = 60:40 (*v*/*v*); (**d**) Ethanol:water = 40:60 (*v*/*v*); (**e**) Ethanol:water = 20:80 (*v*/*v*); (**f**) Ethanol:water = 0:100 (*v*/*v*).

The surface characteristics of camel hair bleached using different concentrations of ethanol during the oxidative bleaching process are shown in Figure 14. It is well-known that the morphology of protein fibers, such as wool and camel hair, is characterized by the scales, which greatly contribute to protecting the protein fibers from damage and affect other essential properties, such as luster and shrinkage [62]. The SEM images demonstrate that the scales on the fibers bleached with the solution composed of 100% ethanol were clear and arranged compactly around each fiber. As the concentration of ethanol in the oxidative bleaching solution was reduced, significant degradation of camel hair was observed. In other words, the cuticle of camel hair was damaged dramatically as the volume of water increased during the oxidative bleaching process.

**Figure 15:**
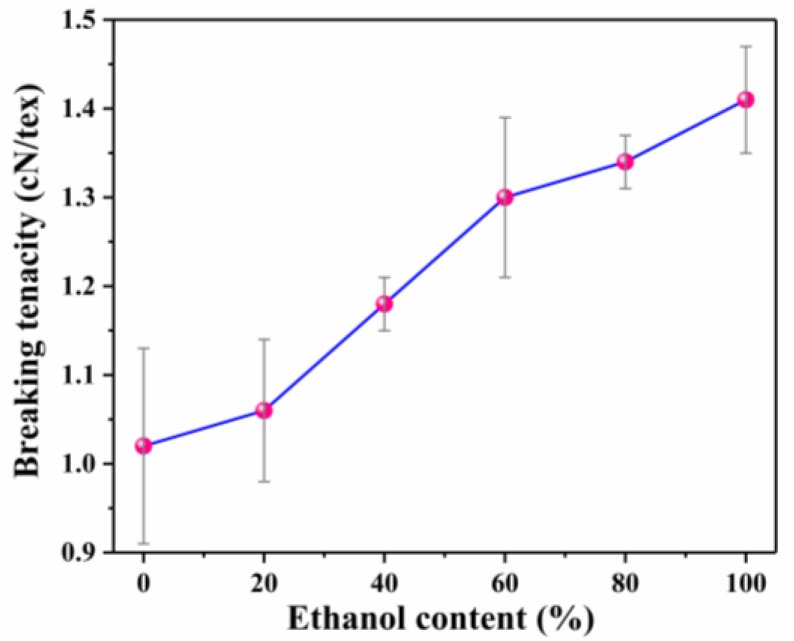
Effects of ethanol content in oxidative bleaching solution on breaking tenacity of camel hair.

To study the influence of ethanol content on mechanical properties of camel hair, the variation of the breaking tenacity of bleached camel hair with the ethanol content is graphically represented in Figure 15. As one can see, a steady increase in breaking tenacity was observed with an increasing volume ratio of ethanol in oxidative bleaching solution. This is in accord with the results from the SEM images in Figure 14. Consequently, the mechanical properties of camel hair can be protected in the presence of ethanol.

Pham et al. [63] reported that contact between hydrogen peroxide and iron-containing minerals (Fe^2+^) may result in the formation of highly reactive and damaging hydroxyl radicals (·OH). This was shown more clearly by Smith et al. [64], who concluded that iron-containing minerals are more likely to activate Fenton reactions, leading to the catalytic decomposition of H_2_O_2_. During this reaction, hydrogen peroxide could form reactive oxygen species, such as hydroxyl radicals (·OH), perhydroxyl radicals (HO_2_·), and superoxide anions (O_2_·^−^). According to the work of Dunford [65], the mechanism of the reaction between Fe^2+^ and H_2_O_2_ has been widely assumed to be as follows.

(4) $${\mathrm{Fe}}^{2+}\;+\;H_{2}O_{2}\to {\mathrm{Fe}}^{3+}\;+\;\cdot \mathrm{OH}\;+\;{\mathrm{OH}}^{-}$$

(5) $${\mathrm{Fe}}^{2+}\;+\;\cdot \mathrm{OH}\to {\mathrm{Fe}}^{3+}\;+\;{\mathrm{OH}}^{-}$$

(6) $$\cdot \mathrm{OH}\;+\;H_{2}O_{2}\to {\mathrm{HO}}_{2}\cdot \;+\;H_{2}O$$

(7) $${\mathrm{HO}}_{2}\cdot \;+\;{\mathrm{Fe}}^{2+}\to {\mathrm{Fe}}^{3+}\;+\;{\mathrm{HO}}_{2}^{-}$$

(8) $$O_{2}\cdot^{-}\;+\;{\mathrm{Fe}}^{3+}\to {\mathrm{Fe}}^{2+}\;+\;O_{2}$$

In our work, there are several possible reasons for the observation of less damage and an enhanced degree of whiteness when ethanol–water mixtures are used during the bleaching process. Firstly, ethanol may accelerate the initial oxidation of ferrous ions during the oxidative bleaching process [66,67]. The effect of ethanol may be interpreted based on the mechanism given below.

(9) $${\mathrm{CH}}_{3}{\mathrm{CH}}_{2}\mathrm{OH}\;+\;\cdot \mathrm{OH}\;+\;{\mathrm{HO}}_{2}\cdot \to {\mathrm{CH}}_{3}\dot{C}\mathrm{HOH}\;+\;H_{2}O\;+\;H_{2}O_{2}$$

(10) $${\mathrm{CH}}_{3}\dot{C}\mathrm{HOH}\;+\;O_{2}\to {\mathrm{CH}}_{3}C\left(\dot{O_{2}}\right)\mathrm{HOH}$$

(11) $${\mathrm{CH}}_{3}C\left(\dot{O_{2}}\right)\mathrm{HOH}\;+\;{\mathrm{HO}}_{2}\cdot \to {\mathrm{CH}}_{3}C\left(O_{2}H\right)\mathrm{HOH}\;+\;O_{2}$$

According to Equations (5), (7), and (9), ethanol molecules will compete with ferrous ions (Fe^2+^) for hydroxyl radicals (·OH) and perhydroxyl radicals (HO_2_·). This would lead to a corresponding reduction in the number and reactivity of the radicals produced. Thus, according to the SEM observations in Figure 14, attack of the fibers by radicals can be decreased to some extent by increasing the concentration of ethanol. Meanwhile, as ferrous ions (Fe^2+^) are preferentially absorbed by the melanin pigments, a strong interaction is formed between them during the mordanting process; thus, the structure of the melanin polymer can be effectively disrupted by the oxidation of ferrous ions. Meanwhile, ferrous ions are more firmly bound to melanin than to keratin. Accordingly, although the reactions shown in Equations (9)–(11) seem to be minor reactions compared with the radical reactions (Equations (4)–(8)), they only occur in melanin, not in keratin, which may also lead to decreased fiber damage. Moreover, ethanol may directly participate in the free radical reaction induced by ferrous iron through Fenton-type reactions, forming hydroxyethyl free radicals as an intermediate from hydroxyl radicals (·OH) and perhydroxyl radicals (HO_2_·) [68,69], thereby causing more damage to melanin as reflected by the observed degree of whiteness (Table 1).

## 4. Conclusions

To protect camel hair and enhance its whiteness degree during bleaching, the fibers were mordanted with ferrous sulfate heptahydrate and then subjected to oxidative bleaching with hydrogen peroxide in a neutral ethanol–water system. This process significantly improved the whiteness degree of the fibers without substantial tenacity loss compared with those of the fibers bleached in aqueous solution. Swelling is crucial for effective mordanting, and increased swelling was observed in the presence of ethanol, likely owing to good permeation of ethanol into the fibers. SEM images showed that ethanol could prevent the fibers from suffering serious damage during the bleaching treatment. Increased modification of the scales was observed when a higher ratio of water was applied during the oxidative bleaching process, whereas treatment in ethanol under neutral conditions made the camel hair less accessible to proteolytic attack. Moreover, changes in the FTIR absorption band corresponding to hydrogen bonding in the camel hair fibers suggested that breakage of these intramolecular hydrogen bonds during the bleaching treatment in aqueous solution was relatively more severe than that during treatment in the ethanol–water system. These findings indicate that the impact of chemical bleaching on the whiteness degree and mechanical properties of camel hair can be well-controlled by the addition of ethanol during the mordanting and oxidative bleaching processes. Further work in this direction is being performed to investigate the effect of this approach on the desirable properties of other natural protein fibers.

## Figures and Tables

**Table 1:** Degree of whiteness and weight loss of camel hair bleached in the ethanol–water and water systems.

Camel Hair	Degree of Whiteness	Whiteness Increase (%)	Weight Loss (%)
Control samples	12.3 ± 0.2	-	-
Samples bleached in the ethanol–water system	32.4 ± 1.5	163.1 ± 0.3	10.1 ± 0.2
Samples bleached in the water system	19.0 ± 0.8	54.1 ± 0.2	9.1 ± 0.1

**Table 2:** Mechanical properties of camel hair bleached in the water and ethanol–water systems.

Camel Hair	Breaking Tenacity (cN/dtex)	Minimum and Maximum of Breaking Tenacity (cN/dtex)	Breaking Elongation (%)
Control sample	1.65 ± 0.19	1.13/2.55	36.18 ± 4.03
Sample bleached in the ethanol–water system	1.24 ± 0.32	0.70/2.12	30.53 ± 11.12
Sample bleached in the water system	0.84 ± 0.22	0.48/1.24	16.74 ± 3.92

**Table 3:** Average values of diameters and swelling ratios for camel hair obtained by the optical method.

System	Swelling Bath	D_b_ (μm)	D_a_ (μm)	R_s_
Water	2 g/L FeSO_4_·7H_2_O Ethanol:water = 0:100 (*v*/*v*)	21.29 ± 0.34	21.62 ± 0.55	1.02 ± 0.03
Ethanol–water	2 g/L FeSO_4_·7H_2_O Ethanol:water = 60:40 (*v*/*v*)	23.15 ± 1.21	24.95 ± 1.34	1.14 ± 0.08
Ethanol–water	2 g/L FeSO_4_·7H_2_O Ethanol:water = 80:20 (*v*/*v*)	23.70 ± 1.41	27.88 ± 1.38	1.20 ± 0.02
